# Changing trends in the management of pediatric distal forearm fractures: a descriptive Danish 20-year nationwide registry study of 175,083 cases

**DOI:** 10.2340/17453674.2025.45057

**Published:** 2026-01-19

**Authors:** Katrine Rønn ABILDGAARD, Per Hviid GUNDTOFT, Stig BRORSON, Bjarke VIBERG

**Affiliations:** 1 Centre for Evidence-Based Orthopedics, Department for Orthopedic Surgery, Zealand University Hospital, Køge; 2 Department of Orthopedic Surgery and Traumatology, Hospital Lillebaelt Kolding, University Hospital of Southern Denmark; 3 Department of Orthopedic Surgery and Traumatology, Aarhus University Hospital, Aarhus; 4 Department of Orthopedic Surgery and Traumatology, Odense University Hospital, Odense, Denmark

## Abstract

**Background and purpose:**

Management of pediatric distal forearm fractures ranges from no or simple immobilization to surgical fixation. Treatment decisions depend on age and fracture severity, but practices vary widely across countries. As surgical intervention has increased internationally, we aimed to investigate national trends in incidence and treatment of pediatric distal forearm fractures in Denmark from 1999–2018

**Methods:**

We conducted a population-based register study of children aged 0–15 with distal forearm fractures (S525 + S526) registered in the Danish National Patient Registry from 1999–2018. Treatments within 1 week of injury were grouped into: non-surgical (no immobilization, soft bandage or cast immobilization), closed reduction and immobilization, and surgical fixation. Procedure codes included closed reduction, open reduction, K-wires, or other fixations such as external fixation, nail, plate, or screws.

**Results:**

There were 175,083 fractures over the 20 years, yielding a mean incidence of 829/100,000/year in children aged 0–15 years. The highest incidences were 1,494/100,000 among 11-year-old girls and 1,720/100,000 among 13-year-old boys. The primary treatment in all age groups was non-surgical treatment, though decreasing from 92% in 1999 to 89% in 2018. The proportion of closed reduction declined from 7% to 2%, while K-wire fixation increased from 1% to 8%. When stratified by age groups, the same trend was seen in all but the 0–3-year-olds.

**Conclusion:**

The overall incidence remained stable during the study period. Non-surgical treatment remained predominant, whereas closed reductions decreased in favor of more K-wire fixations.

Distal forearm fractures are the most common pediatric fractures, accounting for ~25% of cases [1]. Management can be non-surgical (no immobilization, soft bandage or cast immobilization), closed reduction and casting, or surgical with reduction and fixation [2,3]. Torus fractures and Salter-Harris (SH) type I fractures are typically treated with a dorsal splint or soft bandage for 3 weeks with no follow-up [3]. Treatment decisions regarding greenstick, complete, and SH type II–IV fractures depend on degree of displacement (angulation and translation), thresholds varying with age and between countries, hospitals, and surgeons [4]. Awareness of treatment trends is important to standardize pediatric fracture care regarding patient burden and cost-effectiveness.

Reported incidences range from 337 to 784 per 100,000 persons/year, with peaks among 7–10-year-old girls and 10–14-year-old boys [5-7]. Variation between studies reflects different inclusion criteria and registries, making comparison difficult.

Indications for surgery vary across countries. Although non-surgical treatment predominates [2], surgical treatment has increased across all fracture types [8]. Closed reduction may be an alternative when non-surgical treatment is insufficient. Whereas some countries perform closed reduction under sedation in the emergency department [9,10], in other countries, including Denmark and Sweden, it is usually performed under general anesthesia with optional Kirschner wires (K-wires) [11,12]. Yet, a Danish report from 2025 recommends closed reduction under sedation in the emergency department for forearm fractures in 4–15-year-old children, provided safety prerequisites are met [13].

The aim of our study was to report incidence and treatment trends of pediatric distal forearm fractures in Denmark over the last 20 years, stratified by age.

## Methods

### Study design

This population-based register study includes 0–15-year-old children with distal forearm fractures registered in the Danish National Patient Registry (DNPR) from 1999–2018. The study is reported according to RECORD guidelines [14].

### Setting

Denmark has a population of nearly 6 million [15]. All citizens are provided a unique CPR (Central Person Register) number [16], which grants free healthcare and ensures registration of all healthcare contacts in national databases. As treatment is free, the mean distance to hospitals is 20 km [17], and radiographs are only available at hospitals. All fracture treatments are provided in public hospitals; acutely in the emergency rooms and during follow-up in outpatient clinics or general practice, depending on severity.

### Data sources

The DNPR, linked to the Central Person Register, contains data on inpatients since 1977 and outpatients since 1995 [18]. Registration of all diagnoses and procedures is mandatory whenever a person is treated in the public healthcare system, enabling extensive information on health status, medications, and treatments. Data completeness is nearly 100% [15,18], and the Positive Predictive Value (PPV) of primary diagnoses in orthopedic surgery is approximately 83% [18]. Whereas the exact PPV for childhood fractures is unknown, for adult hip, humerus and ankle fractures, it is >90%, 89%, and 95%, respectively [19-21].

### Participants

**Figure 1:**
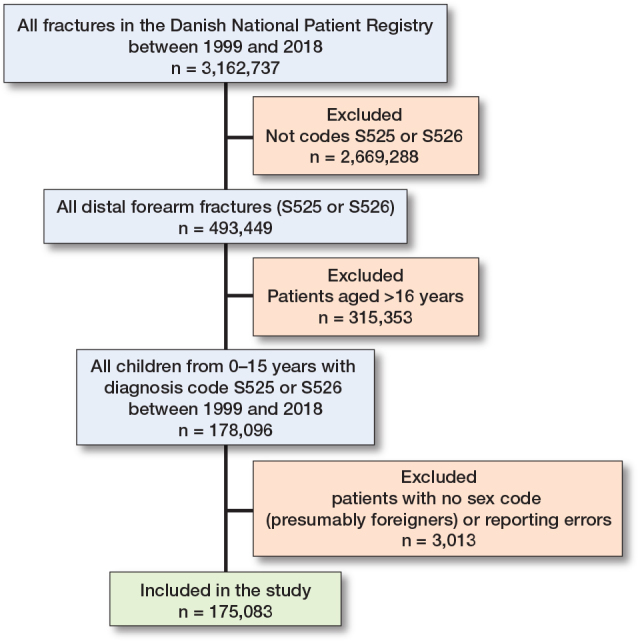
Patient flowchart.

All patients between 0 and 15 years with ICD-10 diagnosis codes for distal radius fractures (S525) and distal radius and ulna fracture (S526) were included. Because these codes encompass all fracture types, from torus to displaced fractures, they are all represented in the dataset (Figure 1).

### Variables

Although the CPR and DNPR are linked, data extraction was performed anonymously. The following data were extracted: sex, age, year of injury, ICD-10 code, and procedure codes. Yearly population counts for each age, defined as survivors by December 31st, were obtained from Statistics Denmark [15]. Fracture incidence was reported for girls and boys overall and within the age groups 0–3, 4–7, 8–11, and 12–15 years. These groups reflect common clinical practice and treatment strategies. Treatment definitions were:

- Non-surgical: no immobilization, soft bandage or cast immobilization only (above- or below-elbow splint or circular cast) within 1 week from injury.
- Closed reduction and immobilization: procedure code for closed reduction and immobilization under general anesthesia within 1 week from injury.
- Surgical fixation: procedure code for closed or open reduction with fixation, including K-wires, or other type of fixation such as external fixation, nail, or plate and screws and immobilization within 1 week from injury.

### Statistics

All statistical analyses and data visualizations were performed using R (R Foundation for Statistical Computing, Vienna, Austria, version 4.3.3).

Descriptive statistics are presented as numbers and percentages. Incidences were calculated as cases per 100,000 persons/year with 95% confidence intervals (CI) (Poisson distribution), based on the number of cases divided by the at-risk population. Treatment proportions were calculated as the number in each treatment category divided by the total number of treatments, with 95% CI (binomial distribution). No formal statistical comparisons between groups or over time were performed, but 95% CIs were compared for potential overlap.

### Data access and cleaning methods

The authors had access to the Danish Health Data Authority’s research database facilities (Forskermaskinen).

### Ethics, data sharing, funding, and disclosures

The author group had full access to data but are not allowed to share the raw data. Some cleaned data can be shared upon request. According to Danish legislation, no ethical approval was needed. The study is part of a PhD project, partly funded by Region Zealand and external funders (King Christian the Tenth’s Foundation, The Ville Heise Grant, and Guildal Foundation). None of the authors received commercial funding. The authors declared no conflicts of interest. Complete disclosure of interest forms according to ICMJE are available on the article page, doi: 10.2340/17453674.2025.45057

## Results

### Incidence

**Figure 2:**
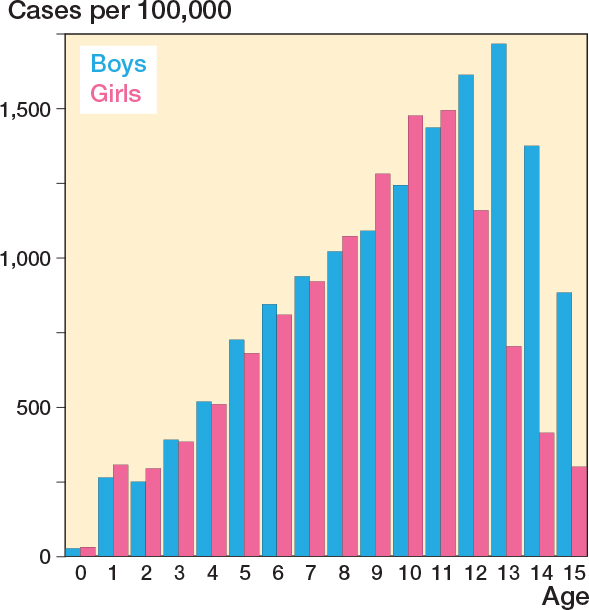
Incidence of distal forearm fractures in 0–15-year-old children.

**Figure 3:**
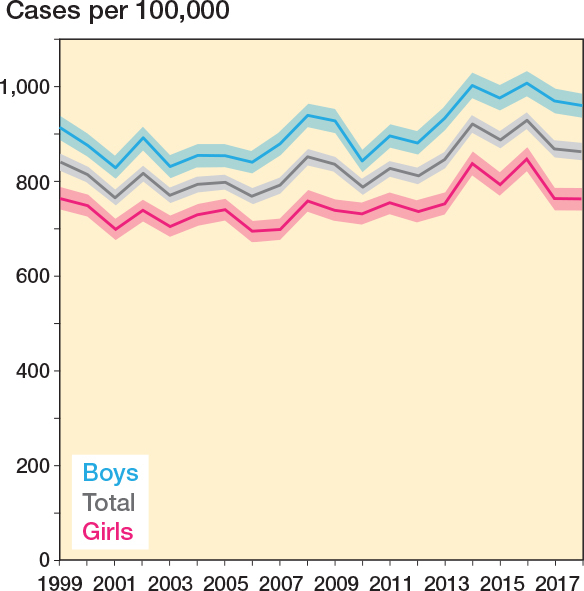
Annual incidence of distal forearm fractures in boys, girls and total.

There were 175,083 fractures, with boys accounting for 56% of cases (n = 97,966). Among the 9–13-year-olds, more than 15,000 fractures were registered in each age group, in total constituting 88,794 (51%) of all fractures (Table 1). The mean annual incidence over the study period was 829/100,000 for children aged 0–15 years (Table 2). Boys had a higher incidence (905/100,000) than girls (749/100,000). For boys, the peak incidence was among 13-year-olds with 1,720/100,000, and for girls, the peak incidence was among 11-year-olds with an incidence of 1,494/100,000 (Figure 2). When assessing the incidence time trends, the incidences showed an upward trend, but with considerable annual variations (Figure 3). The incidence per 100,000 for the total population was 840 (CI 822–858) in 1999 and 863 (CI 845–881) in 2018. For boys, the incidence was 913 (CI 887–939) and 960 (CI 934–987), respectively, and for girls it was 763 (CI 739–787) and 761 (CI 737–788). For the total population as well as boys and girls separately, the CI overlapped.

**Table 1:** Number (counts) of distal forearm fractures in 0–15-year-old children from 1999 to 2018

	Total	1999	2000	2001	2002	2003	2004	2005	2006	2007	2008	2009	2010	2011	2012	2013	2014	2015	2016	2017	2018
Total	175,083	8,575	8,433	8,047	8,690	8,254	8,568	8,646	8,320	8,557	9,202	9,010	8,466	8,825	8,560	8,853	9,566	9,143	9,565	8,938	8,865
Sex																					
Boys	97,966	4,780	4,654	4,474	4,863	4,574	4,733	4,740	4,662	4,875	5,202	5,126	4,635	4,902	4,765	5,009	5,328	5,156	5,314	5,117	5,057
Girls	77,117	3,795	3,779	3,573	3,827	3,680	3,835	3,906	3,658	3,682	4,000	3,884	3,831	3,923	3,795	3,844	4,238	3,987	4,251	3,821	3,808
Age																					
0	374	24	22	28	24	20	20	20	18	17	18	16	19	19	11	17	19	12	13	21	16
1	3,641	232	248	205	242	185	186	213	194	173	177	197	170	173	137	160	148	136	144	155	166
2	3,514	227	199	202	230	179	178	179	215	171	179	165	172	177	141	151	146	151	149	160	143
3	5,038	287	292	262	306	275	249	253	245	248	269	264	251	260	204	232	247	219	221	205	249
4	6,774	359	401	323	348	346	336	367	353	316	330	361	318	298	327	305	367	327	321	353	318
5	9,359	511	502	465	534	412	450	472	492	444	458	454	480	462	449	458	463	476	482	435	460
6	11,104	600	587	505	596	554	559	570	515	538	552	586	487	534	526	598	578	548	615	540	516
7	12,539	705	676	572	653	644	583	609	611	581	593	593	562	625	596	613	705	641	701	640	636
8	14,135	764	697	663	716	689	690	736	602	675	747	694	644	703	672	732	754	753	790	731	683
9	16,005	790	777	773	812	774	798	770	705	765	780	841	769	778	756	792	898	857	885	854	831
10	18,271	835	872	883	927	823	865	925	821	918	987	952	849	940	866	887	1,003	1,033	1,004	918	963
11	19,659	917	909	879	880	948	986	949	986	980	1,134	985	929	1,040	1,030	917	1,087	1,009	1,084	1,043	967
12	18,590	794	828	857	817	835	902	889	889	906	1,036	994	959	940	935	1,029	1,001	1,026	1,043	961	949
13	16,269	659	681	635	739	753	792	763	762	836	910	873	800	813	876	867	947	877	946	850	890
14	11,951	533	456	483	516	498	555	551	571	630	651	638	630	632	624	663	729	620	712	621	638
15	7,860	338	286	312	350	319	419	380	341	359	381	397	427	431	410	432	474	458	455	451	440

**Table 2:** Incidence (cases per 100,000 persons per year) of distal forearm fractures in 0–15-year-old children from 1999 to 2018

Weighted mean		1999	2000	2001	2002	2003	2004	2005	2006	2007	2008	2009	2010	2011	2012	2013	2014	2015	2016	2017	2018
Total	829	840	814	765	817	770	793	798	768	791	852	836	788	827	810	845	923	887	929	869	863
Sex																					
Boys	905	913	876	829	891	831	855	854	840	880	940	928	843	896	881	934	1,003	976	1,007	969	960
Girls	749	763	749	698	739	705	729	740	693	698	759	739	731	754	736	753	838	793	847	762	761
Age																					
0	30	36	33	42	37	31	31	31	28	26	28	25	30	30	19	29	34	21	22	34	26
1	286	341	372	307	358	281	288	327	298	267	269	304	258	272	214	268	252	240	249	262	266
2	274	332	292	302	344	264	270	277	330	263	275	250	264	267	221	234	243	255	259	273	240
3	388	404	427	384	456	410	367	384	379	380	412	405	379	398	307	362	382	363	370	354	423
4	515	504	564	471	508	515	501	542	537	489	506	552	486	448	499	458	572	503	527	586	546
5	704	742	704	653	775	600	669	704	727	675	708	694	732	705	675	698	694	737	735	708	761
6	828	864	852	707	834	802	813	847	769	795	839	904	743	813	802	897	878	817	944	818	836
7	930	1,064	973	827	911	899	843	885	909	868	876	899	865	953	907	933	1,056	969	1,036	975	960
8	1,047	1,163	1,050	952	1,033	959	962	1,063	876	1,005	1,116	1,024	976	1,081	1,023	1,112	1,145	1,123	1,185	1,073	1,037
9	1,184	1,242	1,181	1,161	1,161	1,114	1,109	1,074	1,019	1,113	1,159	1,254	1,133	1,178	1,162	1,204	1,361	1,296	1,311	1,272	1,215
10	1,356	1,363	1,368	1,338	1,387	1,175	1,243	1,285	1,146	1,327	1,434	1,412	1,263	1,383	1,309	1,361	1,522	1,558	1,507	1,352	1,430
11	1,465	1,565	1,480	1,374	1,327	1,414	1,405	1,362	1,370	1,367	1,637	1,428	1,376	1,545	1,513	1,384	1,664	1,524	1,625	1,557	1,419
12	1,393	1,369	1,411	1,391	1,271	1,255	1,342	1,266	1,275	1,257	1,443	1,432	1,388	1,390	1,387	1,508	1,507	1,566	1,566	1,434	1,413
13	1,226	1,164	1,171	1,078	1,194	1,168	1,187	1,134	1,084	1,199	1,261	1,214	1,151	1,175	1,294	1,284	1,385	1,316	1,436	1,270	1,323
14	907	974	803	827	872	802	859	825	849	896	932	882	874	907	900	976	1,076	903	1,061	938	950
15	600	630	521	547	596	537	673	586	509	533	540	566	589	596	587	621	695	672	658	668	661

Stratification by age group likewise suggested a slight increase in incidence among children aged 8–15 years; nevertheless, the CI for all age groups overlapped between 1999 and 2018, indicating that distal forearm fracture incidence remained essentially stable during the study period regardless of age and sex.

### Treatments

**Figure 4:**
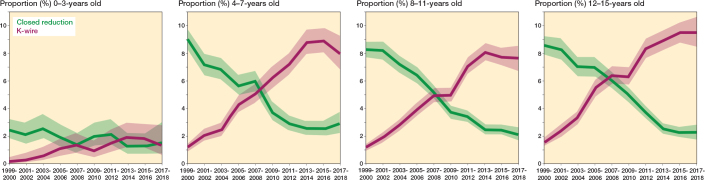
Distribution of closed reduction and K-wire with 95% confidence intervals stratified by age groups 0–3 years, 4–7 years, 8–11 years and 12–15 years, with data summarized in 2-year intervals.

Throughout the investigated period, there were 156,915 (90%) non-surgical treatments, 8,821 (5.0%) closed reductions, 8,749 (5.0%) K-wire fixations, and 598 (0.3%) less commonly used treatments including open reduction, plate and screws, external fixation, and intramedullary nailing (referred to as “other”) (see Supplementary Table). Non-surgical treatment remained most common, though slightly decreasing from 7,864/8,575 (92%) in 1999 to 7,904/8,865 (89%) in 2018, and “other” treatments remained the least common treatment, though slightly increasing from 13/8,575 (0.2%) in 1999 to 52/8,865 (0.6%) in 2018. The second most common treatment changed from closed reduction in 1999 to K-wire fixation in 2018. Closed reduction accounted for 623/8,575 (7.3%) of all treatments in 1999, but only 206/8,865 (2.3%) in 2018, while K-wire fixation accounted for 75/8,575 (0.9%) and 703/8,865 (7.9%), respectively. Stratification by age group revealed the same pattern in all but the youngest children (0–3 years), in whom both treatments accounted for less than 3% of cases and exhibited entirely overlapping CI, limiting the ability to detect any significant change (Figure 4).

## Discussion

We aimed to investigate national trends in incidence and treatment of pediatric distal forearm fractures in Denmark from 1999–2018. We found a largely stable incidence of distal forearm fractures in 0–15-year-old children over a 20-year period in Denmark, in agreement with other studies [5,6,22,23]. The mean incidence of distal forearm fractures was 829/100,000/year with higher incidences in boys than girls and no significant age-group differences. The latter contrasts with Monget et al. [24], who, in the period between 1999 to 2019, reported a shift towards younger children sustaining fractures, possibly due to behavioral changes, with more sedentary behavior among older children and younger children becoming more active, thus increasing the risk of sustaining a fracture.

Peak incidences in the present study align with findings in other studies [2,5,7,22,23]. The most comparable is that by Korup et al. [5], which, like our study, used DNPR data, though with data only from the North Denmark Region and reported a slightly lower incidence (738/100,000/year). They included all children below the age of 18 years, thus the differences likely reflects the marked decline in incidence after the age of 15 years (see Figure 2 and [5,22,23]), while population counts among 16–18-year-old children are roughly equal to younger age groups [15]. Discrepancies may also reflect regional population differences within Denmark; however, this was beyond the scope of the present study.

The most interesting finding was the increase in K-wire fixation across almost all age groups, with a corresponding decline in closed reductions. The point at which K-wire fixation surpassed closed reduction was around 2008–2009. This shift is consistent with the trends observed in pediatric diaphyseal forearm fracture treatment [25], where closed reduction has been largely replaced by intramedullary nailing. However, in contrast to Hansen et al. [25], the observed change in our study is unlikely to be explained by the introduction of new implants as, to the best of our knowledge, no new implants for the treatment of distal forearm fractures have been introduced during the study period, particularly not K-wires, which have been used for decades [26].

The trend is also supported by international studies. A Chinese study reported that, among 0–18-year-old children with upper extremity fractures registered between 2015 and 2019, more children were treated with closed reduction with K-wire fixation (10,050/18,066 [56%]), compared with closed reduction and immobilization (114/18,066 [<1%]) [27]. Monget et al. [24] observed a rise in surgical treatment of pediatric upper and lower extremity fractures in 0–15-year-old children between 1999 and 2019. Likewise, Helenius et al. [8] investigated the incidence of pediatric fractures leading to in-hospital treatment and their treatment during 1997–2006 and reported a 23% increase in the incidence of upper extremity fractures managed in hospital and a 182% increase (from 11 to 31 per 100,000) in surgically treated forearm fractures when closed fracture treatment was excluded. The present study demonstrates a substantially greater increase, with the proportion of K-wire fixations corresponding to a rate ratio of approximately 800%. Notably, Helenius et al. observed an acceleration in surgical treatment toward the end of their study period in 2006. The higher proportions observed in the present study may reflect a continuation of this accelerated trend in the subsequent years.

Our study shows a clear shift toward more surgical treatments despite stable fracture incidence and no introduction of new implants. This raises the question of whether fractures have become more severe over time, necessitating surgical intervention, or there has been a shift in the indications for surgery. Several explanations are possible, and likely multifactorial. Insufficient training in casting techniques may lead surgeons to favor K-wire fixation to avoid re-displacement [28], potentially encouraging a more defensive approach. Strengthening theoretical and clinical training of young orthopedic surgeons in reduction and casting, such as simulation-based training [29], may mitigate this tendency. Another explanation for the observed trend may be a general preference for surgery, reinforced by a predominance of studies concerning surgical treatments and limited evidence supporting non-surgical treatment. Results from 4 RCTs conducted in Denmark [30], Finland [31], USA [32] and the UK [33], are expected to be published in the coming years. These studies challenge current practices by comparing non-surgical treatment with reduction with or without fixation, thereby holding the potential to reduce surgical indications. Along with this upcoming evidence to guide decisions between treatments, future qualitative research could help elucidate the underlying incentives and motivations driving the observed changes in practice.

### Strengths

The primary strength lies in the large sample size and the good representation of the target population. The DNPR is characterized by high completeness in the registration of diagnostic and treatment codes, and despite lack of validation of pediatric forearm fracture coding, it generally demonstrates a high PPV for accurate coding of orthopedic diagnoses.

### Limitations

First, there is a risk of misclassification. Initial diagnoses and treatments are often made in the emergency department by less experienced doctors, increasing the risk of incorrect primary fracture classification. In a survey of 42 doctors with less than 2 years of experience, a high level of uncertainty was reported when interpreting radiographs, underlining the potential for diagnostic inaccuracy in this setting [34]. In addition, the DNPR does not provide clear definitions for diaphyseal versus distal forearm fractures, and miscoding between closely related categories (e.g. radius versus both-bone fractures) may occur. Such misclassification could influence the observed distribution of treatment modalities.

Second, the registry does not include information on fracture displacement, severity, or physeal involvement—factors that are central to treatment decisions. This lack of detail limits our ability to fully interpret observed treatment trends.

As the data was extracted anonymously, it was impossible to validate treatment codes or assess the magnitude of potential misclassification through review of patient records. However, it should be noted that radiographs in Denmark are systematically described or approved by radiologists, which likely reduces the risk of persistent miscoding. Furthermore, Korup et al. [5] validated all 4,316 fractures through manual review of medical records and radiographs, demonstrating that DNPR fracture data can be both accurate and reliable. Given that both their study and the present study are based on DNPR data, and considering the registry’s generally high completeness, positive predictive value, and our large population base, we believe that the relative changes observed in this study are valid despite the limitations.

### Conclusion

We found that distal forearm fracture incidence remained stable throughout the investigated period. We observed a clear shift in treatment patterns over time, characterized by increasing use of K-wire fixation and a corresponding decline in closed reduction. These changes have occurred without obvious reasons or convincing evidence to support one approach over the other.

### Supplementary data

The Supplementary Table is available on the article homepage, doi: 10.2340/17453674.2025.45057

## Supplementary Material



## Notes

KA, SB, PG and BV designed the study. BV and PG had access to Forskermaskinen and extracted data from the DNPR. KA performed statistical analysis and drafted the manuscript, and the results were analyzed with and the manuscript revised by SB, PG and BV.

Handling co-editors: Ilkka Helenius and Robin Christensen

*Acta* thanks Jiri Chomiak and other anonymous reviewers for help with peer review of this manuscript.
