# Supplementary material for: Changing trends in the management of pediatric distal forearm fractures: a descriptive Danish 20-year nationwide registry study of 175,083 cases
Source: Acta Orthop. 2026 Jan 19;97:21–7. doi: 10.2340/17453674.2025.45057 (PMC12816993; doi:10.2340/17453674.2025.45057)
Supplement: Supplementary file 1 [file ActaO-97-45057-s1.pdf]

Supplementary Table 3. Treatment of distal forearm fractures in 0-15-year-old children from 1999 to 2018 in counts (%)

	Total	1999	2000	2001	2002	2003	2004	2005	2006	2007	2008
**Total**	**175,083**	**8,575**	**8,433**	**8,047**	**8,690**	**8,254**	**8,568**	**8,646**	**8,320**	**8,557**	**9,202**
**Age**											
0–3	12,567	770	761	697	802	659	633	665	672	609	643
4–7	39,776	2,175	2,166	1,865	2,131	1,956	1,928	2,018	1,971	1,879	,1933
8–11	68,070	3,306	3,255	3,198	3,335	3,234	3,339	3,380	3,114	3,338	3,648
12–15	54,670	2,324	2,251	2,287	2,422	2,405	2,668	2,583	2,563	2,731	2,978
**Treatment age groups**											
**Non-surgical**	**156,915**	**7,864**	**75,96**	**7,224**	**7,864**	**7,443**	**7,735**	**7,802**	**7,485**	**7,577**	**8,214**
0–3	12,189	746 (96.9)	744 (97.8)	679 (97.4)	779 (97.1)	644 (97.7)	615 (97.2)	641 (96.4)	653 (97.2)	589 (96.7)	624 (97.0)
4–7	35,544	1,984 (91.2)	1,918 (88.6)	1,653 (88.6)	1,929 (90.5)	1,773 (90.6)	1,748 (90.7)	1,821 (90.2)	1,781 (90.4)	1,679 (89.4)	1,722 (89.1)
8–11	61,059	3,015 (91.2)	2,935 (90.2)	2,868 (89.7)	3,005 (90.1)	2,884 (89.2)	2,992 (89.6)	3,040 (89.9)	2,799 (89.9)	2,962 (88.7)	3,269 (89.6)
12–15	48,123	2,119 (91.2)	1,999 (88.8)	2,024 (88.5)	2,151 (88.8)	2,142 (89.1)	2,380 (89.2)	2,300 (89.0)	2,252 (87.9)	2,347 (85.9)	2,599 (87.3)
**Closed reduction**	**8,821**	**623**	**710**	**681**	**653**	**611**	**598**	**558**	**508**	**520**	**508**
0–3	248	23 (3.0)	15 (2.0)	17 (2.4)	21 (2.6)	10 (1.5)	14 (2.2)	20 (3.0)	14 (2.1)	11 (1.8)	9 (1.4)
4–7	2,106	174 (8.0)	212 (9.8)	178 (9.5)	160 (7.5)	134 (6.9)	134 (7.0)	137 (6.8)	125 (6.3)	93 (4.9)	117 (6.1)
8–11	3,532	249 (7.5)	276 (8.5)	283 (8.8)	262 (7.9)	276 (8.5)	254 (7.6)	230 (6.8)	195 (6.3)	220 (6.6)	194 (5.3)
12–15	2,935	177 (7.6)	207 (9.2)	203 (8.9)	210 (8.7)	191 (7.9)	196 (7.3)	171 (6.6)	174 (6.8)	196 (7.2)	188 (6.3)
**K-wire**	**8749**	**75**	**106**	**122**	**162**	**173**	**211**	**266**	**304**	**423**	**448**
0–3	126	1 (0.1)	2 (0.3)	1 (0.1)	2 (0.2)	3 (0.5)	4 (0.6)	4 (0.6)	5 (0.7)	9 (1.5)	10 (1.6)
4–7	2,023	15 (0.7)	30 (1.4)	29 (1.6)	41 (1.9)	45 (2.3)	41 (2.1)	57 (2.8)	64 (3.2)	102 (5.4)	87 (4.5)
8–11	3,282	35 (1.1)	38 (1.2)	44 (1.4)	64 (1.9)	63 (1.9)	85 (2.5)	107 (3.2)	110 (3.5)	147 (4.4)	177 (4.9)
12–15	3,318	24 (1.0)	36 (1.6)	48 (2.1)	55 (2.3)	62 (2.6)	81 (3.0)	98 (3.8)	125 (4.9)	165 (6.0)	174 (5.8)
**Other**	**598**	**13**	**21**	**20**	**11**	**27**	**24**	**20**	**23**	**37**	**32**
0–3	4	0 (0.0)	0 (0.0)	0 (0.0)	0 (0.0)	2 (0.3)	0 (0.0)	0 (0.0)	0 (0.0)	0 (0.0)	0 (0.0)
4–7	103	2 (0.1)	6 (0.3)	5 (0.3)	1 (0.0)	4 (0.2)	5 (0.3)	3 (0.1)	1 (0.1)	5 (0.3)	7 (0.4)
8–11	197	7 (0.2)	6 (0.2)	3 (0.1)	4 (0.1)	11 (0.3)	8 (0.2)	3 (0.1)	10 (0.3)	9 (0.3)	8 (0.2)
12–15	294	4 (0.2)	9 (0.4)	12 (0.5)	6 (0.2)	10 (0.4)	11 (0.4)	14 (0.5)	12 (0.5)	23 (0.8)	17 (0.6)

	2009	2010	2011	2012	2013	2014	2015	2016	2017	2018
**Total**	**9,010**	**8,466**	**8,825**	**8.,560**	**8,853**	**9,566**	**9,143**	**9.,565**	**8,938**	**8,865**
**Non-surgical**	8,013	7,599	8,002	7,633	7,874	8,507	81,28	8,523	7,928	7,904
**Surgical**	997	867	823	927	979	1059	1015	1042	1010	961
**Age**										
0–3	642	612	629	493	560	560	518	527	541	574
4–7	1,994	1,847	1,919	1,898	1,974	2,113	1,992	2,119	1,968	1,930
8–11	3,472	3,191	3,461	3,324	3,328	3,742	3,652	3,763	3,546	3,444
12–15	2,902	2,816	2,816	2,845	2,991	3,151	2,981	3,156	2,883	2,917
**Treatment age groups**										
**Non-surgical**	**8,013**	**7,599**	**8,002**	**7,633**	**7,874**	**8,507**	**8,128**	**8,523**	**7,928**	**7,904**
0–3	625 (97.4)	595 (97.2)	608 (96.7)	475 (96.3)	539 (96.2)	541 (96.6)	501 (96.7)	516 (97.9)	518 (95.7)	557 (97.0)
4–7	1,760 (88.3)	1,649 (89.3)	1,730 (90.2)	1,720 (90.6)	1,746 (88.4)	1,865 (88.3)	1,753 (88.0)	1,873 (88.4)	1,728 (87.8)	1,712 (88.7)
8–11	3,110 (89.6)	2,883 (90.3)	3,171 (91.6)	2,964 (89.2)	2,966 (89.1)	3,329 (89.0)	3,252 (89.0)	3,365 (89.4)	3,163 (89.2)	3,087 (89.6)
12–15	2,518 (86.8)	2,472 (87.8)	2,493 (88.5)	2,474 (87.0)	2,623 (87.7)	2,772 (88.0)	2,622 (88.0)	2,769 (87.7)	2,519 (87.4)	2,548 (87.4)
**Closed reduction**	**469**	**366**	**329**	**293**	**289**	**255**	**207**	**216**	**221**	**206**
0–3	9 (1.4)	12 (2.0)	13 (2.1)	7 (1.4)	16 (2.9)	6 (1.1)	8 (1.5)	4 (0.8)	10 (1.8)	9 (1.6)
4–7	119 (6.0)	75 (4.1)	67 (3.5)	48 (2.5)	66 (3.3)	59 (2.8)	47 (2.4)	52 (2.5)	52 (2.6)	57 (3.0)
8–11	174 (5.0)	129 (4.0)	120 (3.5)	123 (3.7)	106 (3.2)	103 (2.8)	83 (2.3)	94 (2.5)	87 (2.5)	74 (2.1)
12–15	167 (5.8)	150 (5.3)	129 (4.6)	115 (4.0)	101 (3.4)	87 (2.8)	69 (2.3)	66 (2.1)	72 (2.5)	66 (2.3)
**K-wire**	**504**	**471**	**465**	**601**	**659**	**766**	**764**	**778**	**748**	**703**
0–3	8 (1.2)	5 (0.8)	7 (1.1)	11 (2.2)	5 (0.9)	13 (2.3)	8 (1.5)	7 (1.3)	13 (2.4)	8 (1.4)
4–7	113 (5.7)	118 (6.4)	118 (6.1)	127 (6.7)	154 (7.8)	180 (8.5)	182 (9.1)	182 (8.6)	184 (9.3)	154 (8.0)
8–11	177 (5.1)	170 (5.3)	163 (4.7)	226 (6.8)	248 (7.5)	298 (8.0)	301 (8.2)	287 (7.6)	279 (7.9)	263 (7.6)
12-15	206 (7.1)	178 (6.3)	177 (6.3)	237 (8.3)	252 (8.4)	275 (8.7)	273 (9.2)	302 (9.6)	272 (9.4)	278 (9.5)
**Other**	**24**	**30**	**29**	**33**	**31**	**38**	**44**	**48**	**41**	**52**
0–3	0 (0.0)	0 (0.0)	1 (0.2)	0 (0.0)	0 (0.0)	0 (0.0)	1 (0.2)	0 (0.0)	0 (0.0)	0 (0.0)
4–7	2 (0.1)	5 (0.3)	4 (0.2)	3 (0.2)	8 (0.4)	9 (0.4)	10 (0.5)	12 (0.6)	4 (0.2)	7 (0.4)
8–11	11 (0.3)	9 (0.3)	7 (0.2)	11 (0.3)	8 (0.2)	12 (0.3)	16 (0.4)	17 (0.5)	17 (0.5)	20 (0.6)
12–15	11 (0.4)	16 (0.6)	17 (0.6)	19 (0.7)	15 (0.5)	17 (0.5)	17 (0.6)	19 (0.6)	20 (0.7)	25 (0.9)
